# Analysis of Balance during Functional Walking in Stroke Survivors

**DOI:** 10.1371/journal.pone.0166789

**Published:** 2016-11-17

**Authors:** Fokke B. van Meulen, Dirk Weenk, Edwin H. F. van Asseldonk, H. Martin Schepers, Peter H. Veltink, Jaap H. Buurke

**Affiliations:** 1 Biomedical Signals and Systems, MIRA-Institute for Biomedical Technology and Technical Medicine, University of Twente, Enschede, the Netherlands; 2 Biomechanical Engineering, MIRA-Institute for Biomedical Technology and Technical Medicine, University of Twente, PO Box 217, 7500 AE, Enschede, the Netherlands; 3 Xsens Technologies B.V., Enschede, the Netherlands; 4 Roessingh Research and Development, Roessingh Rehabilitation Center. Enschede, the Netherlands; University of Tuebingen, GERMANY

## Abstract

**Background:**

An important objective of rehabilitation care is to regain adequate balance function to safely ambulate in community. However, in rehabilitation practice, it remains unclear if a stroke survivor functionally recovers by restitution or by learning to compensate for the lack of restoration of body function. Aim of this study is to propose and evaluate methods for the objective evaluation of balance during functional walking in stroke survivors.

**Methods:**

Stroke survivors performed twice a Timed “Up & Go” (TUG) test. Ground reaction forces and position changes of both feet were measured using instrumented shoes and used to estimate the position of the center of mass (CoM). Balance control and efficiency metrics were defined to evaluate functional walking under variable conditions. Metrics were corrected based on the instantaneous velocity direction of CoM. Intra- and inter-participant variations for different phases of the TUG test were examined. Metrics were related to the Berg balance scale (BBS).

**Results:**

Participants with higher BBS scores show a more efficient walking pattern. Their walking velocity and walking direction is less variable and they are more frequently unstable when walking in a straight line or when turning. Furthermore, the less affected participants are able to move their CoM more towards their affected side.

**Discussion:**

We developed and demonstrated a method to assess walking balance of stroke survivors. System design and evaluation methods allow balance evaluation during functional walking in daily life. Some presented metrics show correlations with BBS scores. Clear inter- and intra-patient variations in metric values are present that cannot be explained by BBS scores, which supports the additional value of the presented system. Presented methods may be used for objective evaluation of restitution and compensation of walking balance and have a potential application in individual evidence-based therapy.

## Introduction

Stroke survivors are frequently confronted with an impaired balance function [1]. They have a large risk of falling at home after they have been discharged from a rehabilitation center [2–5]. Therefore, an important objective of rehabilitation care is to regain adequate balance function to safely ambulate in community. During rehabilitation, balance while standing as well as during walking is frequently assessed using standardized clinical tests to predict functional performance [6, 7]. Although the assessed functional recovery of walking balance might be quite substantial, little is known about the underlying mechanisms that contribute to the process of recovery [8]. Current clinical tests evaluate walking balance on an activity level by describing the ability to complete a task and the time needed to complete a task. These tests do not specifically evaluate balance on the level of body function, as these levels are described in the international classification of functioning (ICF) [9, 10]. By evaluating walking balance on an activity level only, it remains unclear if an individual stroke survivor functionally recovers by restitution or by learning to compensate for the lack of restoration of body function. To get these insights in walking balance and body function, an objective assessment of body function during walking is required.

Especially during functional tasks, stroke survivors are confronted with near falls while making special maneuvers during walking [2] or transfers [4, 5]. Furthermore, nearly every activity during daily living includes variable walking, i.e., variable walking speed, changing walking directions, stepping sideways, transfers etc. [7]. These variable walking patterns are more challenging than straight line walking for those with an impaired walking balance [11, 12]. When turning for instance, stroke survivors require a longer time, make more steps and stagger during turning [12, 13]. Assessment of walking balance should include these more variable and challenging conditions, which can be achieved by assessing walking balance under daily life conditions.

Current research and rehabilitation practice is mostly focused on the instrumented assessment of straight line walking. Sensing systems used for the evaluation of walking and mechanisms of walking balance are lab-based and particularly suited for assessing kinematic and kinetic metrics of walking in a straight line. Examples of such lab-based systems are optical marker systems with or without the use of force plates [11, 14], instrumented treadmills [11] or instrumented walkways [15]. Constraints of those systems are limited measurement space to evaluate kinematics and limited force sensing to reconstruct full kinetics of multiple consecutive steps. For the objective evaluation of balance over multiple strides and during turns in a lab or in a daily life setting, wearable sensing systems may be a more suitable solution to overcome these constraints [7, 16]. We developed a concept of a wearable sensing system to evaluate walking in a daily life setting. By fusing inertial and ultrasound sensor data, an accurate full three dimensional (3D) reconstruction of foot movements can be made [17]. The reconstruction of foot movements was combined with force sensing to enable the evaluation of kinematics and kinetics of every step. In a previous study, we were able to estimate metrics which describe kinematics and kinetics of stroke patients who are walking in a straight line [18]. In the current study, the previously developped sensing system is used for the objective evaluation of balance function during functional walking in stroke survivors. For actual use in a daily life setting, the sensor system still needs to be implemented in shoes worn during daily life, in a minimal obtrusive manner [18, 19].

The aim of this study is to objectively evaluate balance during functional walking in individual stroke survivors. Metrics that describe walking balance and efficiency at the levels of activity and body function are presented and were evaluated under a controlled condition, in which stroke survivors with varying levels of walking function completed a structured daily life task. All metrics were derived from kinematic and kinetic data acquired with a wearable shoe-based sensing system and related to the level of balance function assessed using the frequently used clinical assessment scale, the Berg Balance Scale (BBS).

## Materials and Methods

### Ethical approval

Experiments described in this manuscript, were part of a larger protocol approved by the local medical ethics committee (METC Twente) and registered in the Dutch Trial Registry (NTR3636). All participants signed written informed consent before participating.

### Measurement setup

**Fig 1:**
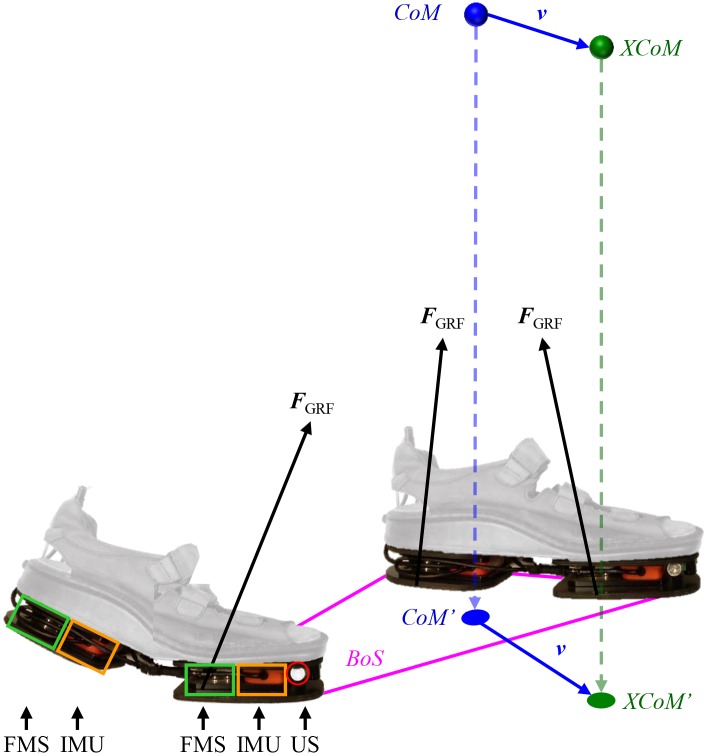
Measurement setup. In this study, Xsens ForceShoes^™^ were used to measure the ground reaction forces (***F***_*GRF*_) and to estimate the position of the base of support (BoS), the body center of mass (CoM) and the extrapolated center of mass (XCoM), which is in the direction of the center of mass velocity (***v***). The instrumented shoes contain one inertial measurement unit (IMU, orange rectangle) and one force/moment (FMS, green rectangle) sensor per heel and forefoot segment. Near the IMU in the forefoot an ultrasound transducer (US, red circle) was mounted in each shoe and is pointing inwards to ensure line of sight. The ground projections of the CoM and XCoM are respectively presented by CoM’ and XCoM’.

A pair of Xsens ForceShoes^™^ (Xsens Technologies B.V., Enschede, The Netherlands) additionally equipped with ultrasound sensors was used to acquire full 3D kinematic and kinetic data of both feet. Each instrumented shoe was equipped with two 3D force/moment sensors and two 3D inertial measurement units (IMU), positioned on the heel and forefoot segments and an ultrasound sensor attached to the forefoot segment of the shoe (see Fig 1). Data of the IMUs and force/moment sensors were collected with a sample frequency of 50 Hz. Distance between both feet was estimated using the ultrasound sensors at an update rate of 13 Hz. Methods described and validated by Weenk, et al. [17] and Schepers, et al. [20] were used to respectively estimate relative feet positions and the position of the center of mass (CoM) during standing and walking.

The instrumented shoes are sandals with Velcro^®^ straps for tightening or untightening the sandals to create the best fit. The instrumented shoes were available in two sizes: EU size 40 and 44. Depending on the foot size of the participant the right size of sandals was selected. The time required to ensure that the wearable sensing system is ready for use was less than five minutes, including donning the instrumented shoes and performing a required zero-force calibration of the force sensors as well as a static calibration of the ultrasound sensors [17].

### Evaluation of dynamic balance

Ensuring adequate balance of the human body during standing and walking is a complex task. A commonly used method to describe balance during standing and walking is the dynamic model of the inverted pendulum [21], which assumes that the whole-body can be described as a point mass situated at the top of the pendulum (center of mass, CoM). To remain in balance during standing and walking, the CoM should remain above the base of support (BoS). However, a CoM position above the BoS does not imply the human body is adequately balanced. Besides the position of the CoM, the velocity of the CoM is of importance as well. Depending on the direction of the velocity of the CoM, the CoM falls towards a certain direction. The extrapolated CoM (XCoM) position is a variable which relates the CoM position and CoM velocity (***v***) [21, 22], as in the following equation:

$$XCoM=CoM+\frac{v}{\omega_{0}}$$ (1)

with $\omega_{0}=\sqrt{g/l_{0}}$, in which *g* is the gravitational acceleration and *l*_0_ is the vertical CoM position.

Depending on the position of the ground projection of the XCoM (XCoM’) relative to the BoS (see Fig 1), different strategies such as muscle activation or stepping can be used to prevent that the body falls to the ground. If the XCoM’ is inside the BoS, muscle activation can be used to influence the size and direction of the velocity of the CoM. When the XCoM’ is outside the BoS, muscle activation only is no longer enough to remain balanced. An extension of the BoS by stepping towards the XCoM’ is necessary to ensure that the inverted pendulum (i.e., human body) is not falling.

For the objective evaluation of mechanisms of functional walking during different behavior, metrics which describe the position and velocity of the CoM relative to both feet (i.e., base of support) are necessary. These metrics should be suitable for the assessment of straight line walking, as well as variable walking. During variable walking, the walking direction changes over time. In contrast with straight line walking or treadmill walking, a constant walking direction cannot be assumed. Therefore it is required to redefine the direction of walk during each step. This allows to estimate metrics in an anterior-posterior and medial-lateral direction.

#### Defining walking direction during functional walking

**Fig 2:**
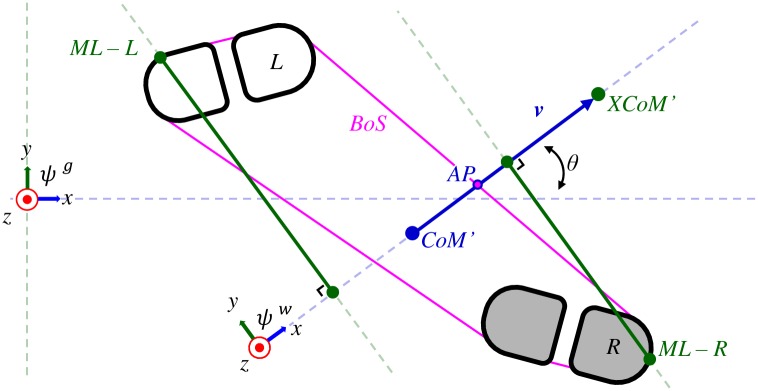
Definitions of measurement frames and balance metrics. Top-down view of left and right shoe during a double support phase in a global frame (*ψ*^*g*^). The blue and green dots represent respectively the ground projections of CoM and XCoM. The blue arrow is the horizontal representation of the velocity vector of CoM (***v***), denoted by CoM’ and XCoM’. In white the left shoe, forefoot (marked with L) and heel. In grey the right shoe, forefoot (marked with R) and heel. Base of support (BoS), is purple enclosed. Margin of stability in anterior-posterior direction is defined as the distance between points AP and XCoM’. Margin of stability in medial-lateral direction for the right and left side are respectively defined as the distance between the *x*-axis of *ψ*^*w*^ and points ML-R (MoS_*r*_) and ML-L (MoS_*l*_) in the direction of the *y*-axis of *ψ*^*w*^. Angle *θ* is the walking direction represented in the global reference frame (i.e., angle between *x*-axis of *ψ*^*w*^ *x*-axis of *ψ*^*g*^).

We use the direction of the velocity of CoM to define the walking direction [22, 23]. By differentiating the position of the CoM over time, the size and direction of velocity of the CoM can be estimated. A moving average filter with a window of 9 samples is applied for smoothing possible abrupt changes of the velocity vector as consequence of differentiating the CoM positions. The velocity vector of the CoM is used to define a local reference frame (*ψ*^*w*^). The *x*-axis of this reference frame is defined in the direction of the velocity of the CoM (***v***), i.e., instantaneous walking direction, the *z*-axis is defined pointing vertically upwards and the *y*-axis is perpendicular to the *x*- and *z*-axis in a right-handed fashion (see Fig 2).

#### Efficiency of walking related to variation of CoM velocity

The direction of CoM velocity, describes the walking direction in a global frame (*ψ*^*g*^). The global reference frame is based on the walking direction during the structured daily life task, such that the positive *x*-axis is in the direction from the starting position to a turn marker three meters away, positive *z*-axis in a vertical upwards direction and *y*-axis perpendicular to the *x*- and *z*-axis in a right-handed fashion. The angle between the estimated walking direction and the *x*-axis of the global reference frame is a variable which describes the walking direction over time (see Fig 2, angle *θ*). The magnitude of the three dimensional CoM velocity describes the size of the velocity vector of the CoM.

Furthermore, direction and size of the CoM velocity can be used to describe the momentum and efficiency of walking. Assuming a constant body mass, any change in direction and size of the CoM velocity is associated with acceleration or deceleration, requiring a mechanical impulse that modifies momentum and kinetic energy of CoM. For a continuous walking pattern, smaller changes in direction and magnitude of the CoM velocity results in a more efficient way of walking [24]. By integrating the resultant forces (***F***) of a certain interval (from *t*_1_ to *t*_2_), the integrated impulse magnitude (*J*) of the interval can be calculated:

$$J=\int_{t_{1}}^{t_{2}}\;||\;F\;||\;dt$$ (2)

In this equation ***F*** can be replaced by the participant’s mass (*m*) times the acceleration vector of the CoM (***a***) according to Newton’s second law of motion. A division of *J* by the average velocity ($\bar{v}$) times the participant’s mass (*m*) results in a normalized impulse ($\hat{J}$, a dimension less metric):

$$\hat{J}=\frac{\int_{t_{1}}^{t_{2}}\;||\;a\;||\;dt}{\bar{v}}$$ (3)

A more continuous walking pattern implies smaller variations of the walking velocity, which means that small changes in acceleration of CoM will result in lower values of $\hat{J}$.

#### BoS and the margin of stability

The margin of stability is the distance between XCoM’ and BoS [21] in the horizontal plane. The BoS is the area underneath one foot in case of single support, or the area underneath both feet including the area between both feet in case of double support. In case one foot is in the air, a virtual BoS (vBos) can be defined as the area of the stance foot, the projection of the swing foot on the ground and the area between both, based on two assumptions. First, the foot of the swing leg can momentaneously be positioned on the ground. Second, the CoP can instantaneously be placed everywhere within the vBoS.

To differentiate between stability in a forward or lateral direction, the margin of stability will be divided into two components. First, the anterior-posterior margin of stability (see Fig 2, line between AP and XCoM’) is defined as the distance between XCoM’ and (v)BoS in the direction of the walking velocity. The forward margin of stability (MoS_*ap*_) is positive when the XCoM’ is outside the (v)BoS and negative when the XCoM’ is within the (v)BoS. A positive forward margin of stability is required for forward progression at an adequate speed during straight line walking. For each part of a trial the percentage of time a participant has a positive margin of stability can be estimated as well as a distribution of the MoS_*ap*_ when standing on the left or right leg. Second, the lateral margin of stability is defined as the maximum distance between the velocity vector and the lateral borders of (v)BoS, defined by the positions (or projections) of the right or the left shoe (see Fig 2, the distance between the *x*-axis of *ψ*^*w*^ and points ML-L and ML-R, along the *y*-axis of *ψ*^*w*^). The lateral margin of stability is a positive value and can be estimated for the left (MoS_*l*_) as well as the right shoe (MoS_*r*_). Asymmetry in the lateral margin of stability is estimated by:

$$\text{Asymmetry}\;{\text{MoS}}_{ml}=\frac{{\text{MoS}}_{l}-{\text{MoS}}_{r}}{{\text{MoS}}_{l}+{\text{MoS}}_{r}}$$ (4)

The Asymmetry MoS_*ml*_ is varying between -1 and 1 in which a positive Asymmetry MoS_*ml*_ indicates a larger lateral margin of stability on the left side than on the right side, a negative Asymmetry MoS_*ml*_ indicates the opposite and a zero value indicates an lateral margin of stability which is equal on both sides.

### Experimental protocol

All metrics were evaluated under controlled conditions, in which stroke survivors with varying levels of clinically assessed balance function completed a structured daily life task. Therefore the Timed “Up & Go” (TUG) test [25] was selected. This study was part of a larger protocol which was previously described in [18, 26].

All participants were recruited from patients of the Roessingh Rehabilitation center, located in Enschede, the Netherlands. The treating physicians screened potential participants and performed the inclusion of stroke survivors. Inclusion criteria were age between 35 and 75 years, a hemiparesis as a result of a single unilateral ischemic or hemorrhagic stroke which is diagnosed at least six months before measurement. Exclusion criteria were a medical history with more than one stroke, the presence of any other disorder that would prevent the participant from being able to complete a walking test without any assistance. Participants who demonstrated an inability to follow instructions or to answer questionnaires were excluded as well.

After receiving verbal instructions only, participants performed a TUG test twice, while wearing the instrumented shoes. The use of a walking aid was prohibited to ensure a complete reconstruction of the ground reaction force. During a TUG test participants raise up from a chair, walk 3 meters, turn around a marker (180 degree turn) and walk back again to the chair and get seated. In clinic, the TUG test is used to assess balance functioning on an activity level and the outcome of this assessment is the time needed to complete the test. The outcome of the TUG test is a good predictor of falls risk and measure for change in mobility in acute and chronic stroke patients [27–30]. Besides the instrumented TUG test, each participant’s balance capacity was assessed using the frequently used clinical assessment test, being the Berg balance scale (BBS). The BBS consists of 14 sub-items in which balance is evaluated while performing different tasks, all sub-items were rated from 0 up to 4 on a ordinal scale (total score ranging from 0–56) [31]. A lower total score is related to a more affected balance capacity and walking function, and a higher risk of falling [2, 3]. Furthermore, all participants’ comfortable walking speed while walking in a straight line is assessed using a 10 meter walk test [32]. All assessments were performed by the same technical physician who has adequate clinical expertise to perform the assessment. During all assessments, participants wore the instrumented shoes.

### Data processing

Data was selected from the first double support phase following the first step after the participant raises up from the chair until the double support phase before the participant starts turning to get seated again. Three phases of the TUG test were defined, the turning phase and the straight line walking phases prior and after the turning phase. The turning phases were manually selected, based on visualized reconstructions of kinematic data. Turns were defined as the periods from the last double support phase before a turn step and the first double support phase after the last turn step [12, 14, 33]. That is, the last double support phase before angle *θ* starts changing towards 180 degrees, till the double support phase after which an angle *θ* of more than 180 degrees has been reached. Metrics will be presented for the first phase of straight line walking and the turning phase. At the end of the third phase of a TUG test (i.e., straight line walking after the turn), participants had to sit down again. Participants used different strategies to prepare for getting seated. Because of this variation, the third phase is not further analyzed.

All kinematic and kinetic data of the instrumented shoes (including ultrasound modules) were processed offline and analyzed using MATLAB^®^ (MathWorks Inc., Natick, MA, USA). Metrics were estimated for each phase of the TUG test and finally averaged over both trials of each participant. Linear regression analysis was performed to estimate correlation values between described metrics and the BBS scores. Correlation values with *p* < 0.05 have been marked as significant.

## Results

Fifteen stroke survivors were recruited of which ten were included in this study and so five dropped out because of the following reasons. Data of four participants were not used due to the following technical reasons. Of two participants data were not fully recorded because of a broken cable during the session or sensors that were not properly functioning. Kinematic data of two other participants could not be reconstructed because no proper synchronization of the IMU and ultrasound sensor data was regained and due to a wrong estimation of initial filter states [17]. Finally, only one of the remaining participants had a right affected side and is therefore excluded as well. Remaining ten participants (seven males and three females) with an average age of 63.2 (SD ± 8.9) years, 2.6 (SD ± 2.0) years post stroke. All participant-specific information is reported in Table 1.

**Table 1:** General participant characteristics.

ID^1^	Gender	Age^2^	Post^3^	Dominant side	Affected side	Weight^4^	Height^5^	BBS^6^	TUG^7^	10M^8^	Walking aid^9^
1	M	54	2.9	R	L	109	1.74	35	24.8	0.43	St, AFO
2	M	69	4.0	R	L	96	1.90	42	18.2	0.62	-
3	F	67	3.3	R	L	80	1.62	43	19.5	0.54	St
4	M	75	1.6	R	L	88	1.72	45	17.4	0.60	St
5	F	55	1.4	R	L	87	1.68	49	16.3	0.74	-
6	M	70	7.4	R	L	94	1.74	52	12.2	0.76	-
7	M	65	1.3	R	L	92	1.86	52	13.1	0.94	OS
8	M	70	1.2	L	L	99	1.81	52	11.0	0.91	-
9	M	47	1.8	R	L	88	1.73	54	11.9	0.95	-
10	F	60	0.7	R	L	74	1.65	55	8.3	1.28	-
Mean (±SD):		63.2 (8.9)	2.6 (2.0)			91 (9.8)	1.74 (0.09)	48 (6.4)	15.2 (4.9)	0.78 (0.25)	

^1^Participant identification number (participants are ranked from a low to high BBS score.).

^2^in years.

^3^years post stroke.

^4^in kilograms.

^5^in meters.

^6^Berg Balance Scale score (0-56 points).

^7^Average time needed to complete the Timed “Up & Go” test, in seconds.

^8^Average walking speed during a 10 meter walk test, in meters per seconds.

^9^Use of walking aid during activities of daily living: St = Stick, AFO = Ankle foot orthosis, OS = Orthopedic Shoes.

**Fig 3:**
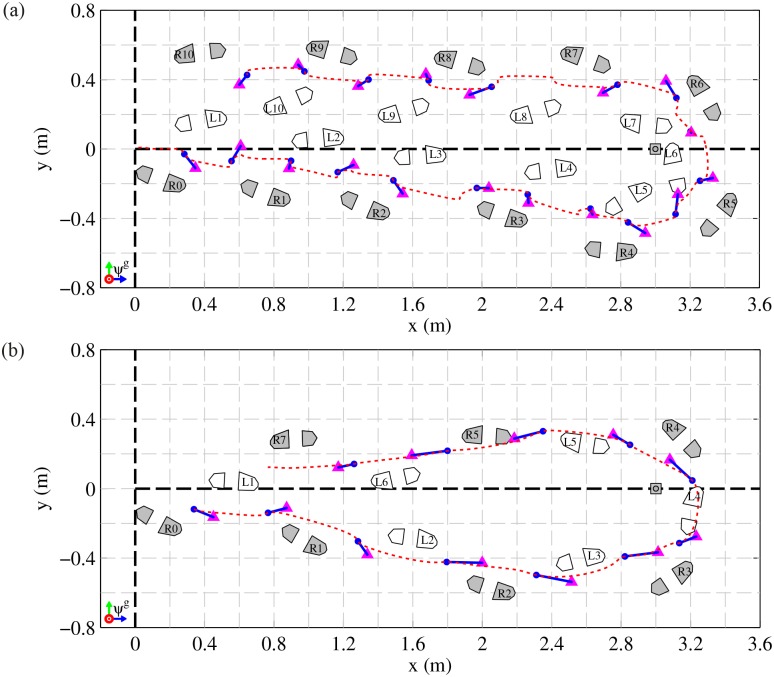
Top-down view of steps made during a TUG test. (a) Participant #3, left side affected, turn from L4-R4 to L7-R7, BBS: 43/56, TUG 19.5 s. (b) Participant #10, left side affected, turn from R2-L3 to L5-R5, BBS: 55/56, TUG 8.3 s. Both participants started at the origin of the graphs and walked around a turn marker (coordinate (0,3)) 3 m away from the starting position. Left (L1, L2, etc.) and Right (R1, R2, etc.) step numbers are indicated in the forefoot segment of the shoe. Blue dots and pink triangles respectively represents the CoM’ and XCoM’ position at the first sample of a double stance phase. The blue line between CoM’ and XCoM’ indicates the direction of the CoM velocity (from CoM to XCoM) just at the beginning of each double support phase, i.e., instantaneous walking direction or *x*-axis of the local reference frame *ψ*^*w*^. The dashed red line is the trajectory of the CoM’ during the selected part of the TUG test.

Fig 3a and 3b show a top-down view of the kinematic reconstruction of the steps made by two participants (#3 and #10 respectively) while performing a single TUG test. Both participants have different scores on the clinical assessment scales. Participant #3 who has a BBS score of 43, needed more time to complete the TUG test and made more steps to complete the test than participant #10 who has a BBS score of 54.

**Fig 4:**
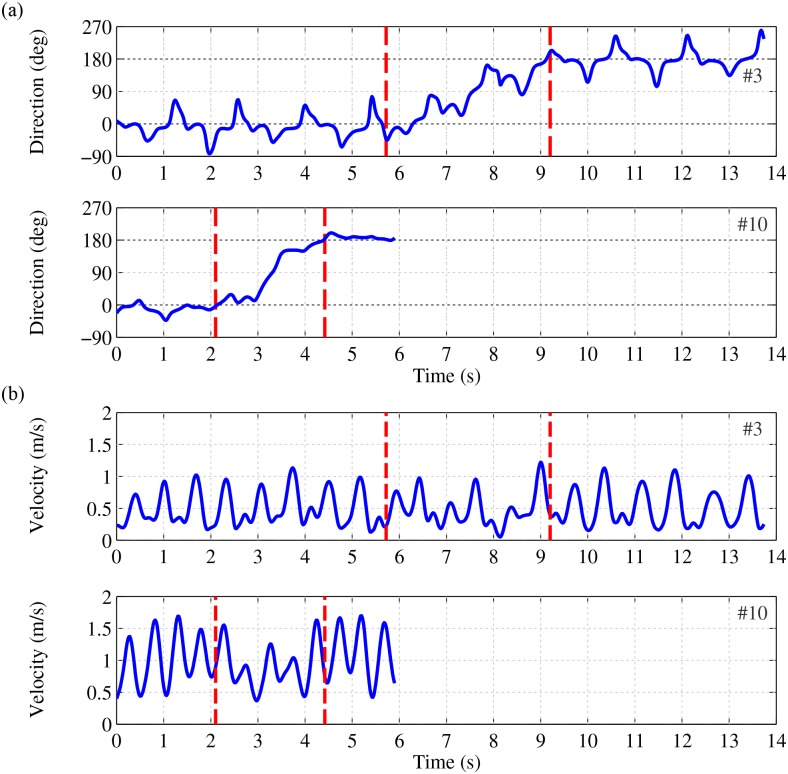
Velocity of CoM while performing a single TUG test. (a) Direction of velocity of CoM relative to global frame (angle *θ*). (b) Magnitude of three dimensional velocity of CoM. Participant #3 (first and third graph) and #10 (second and fourth graph). The red vertical lines indicate the begin and end moments of the turn phase, after and prior to a straight line walking phase, respectively. Metrics were evaluated till the second red line.

**Fig 5:**
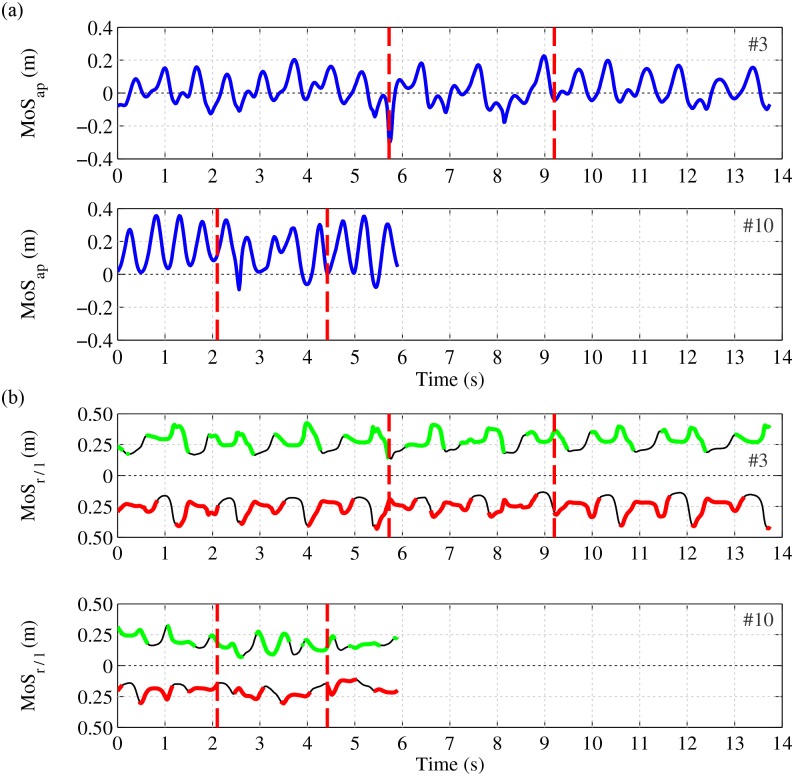
Margin of stability while performing a single TUG test. (a) Anterior-posterior margin of stability, the distance XCoM’ to border of (v)BoS, in direction of velocity of CoM. The value is positive when the XCoM’ position is outside the (v)BoS and negative when the XCoM’ position is inside the (v)BoS. (b) Medial-lateral margin of stability, at each moment the maximum distance between a shoe and the vector between CoM’ and XCoM’ (*x*-axis of *ϕ*^*w*^). The green line (in both graphs the upper thick line) indicates the maximum distance of the left shoe (affected side) to the *x*-axis of *ϕ*^*w*^ (MoS_*l*_), while the foot is in contact with the ground. The red line (in both graphs the lower thick line) indicates the maximum distance of the right shoe to the *x*-axis of *ϕ*^*w*^ (MoS_*r*_), while the foot is in contact with the ground. The black lines represent the distance between the shoes and *x*-axis of *ϕ*^*w*^ when the foot is in swing phase. Participant #3 (first and third graph) and #10 (second and fourth graph). The red vertical lines indicate the begin and end moments of the turn phase, after and prior to a straight line walking phase. Metrics were evaluated till the second red line.

Figs 4 and 5 show more detailed information of the measurements as presented in Fig 3. Fig 4a shows the angle *θ*, i.e., instantaneous walking direction, in the global reference frame for both participants. During the first three meters of straight line walking this angle is on average zero, during the turn it changes towards 180 degrees and at the second part of straight line walking the angle is on average 180 degrees. Walking direction in participant #3 (upper graph of Fig 4a) shows more variability than participant #10 (lower graph of Fig 4a). Fig 4b shows the magnitude of CoM velocity which describes the size of the velocity vector of the CoM. This magnitude is low during double support phases but increases as result of a push off. Both participants show a decrease in average walking velocity while turning (phase between both red dashed lines). A pattern of varying velocity and direction of walking results in a less efficient walking pattern and thereby requires a higher normalized impulse.

Fig 5 shows the stability margins of both participants. In which Fig 5a shows the margin of stability in the anterior-posterior direction, i.e., the distance between XCoM and (v)BoS in the direction of the walking. At moments of a negative margin of stability, the XCoM is inside the (v)BoS, the participant is dynamically stable and no additional step is needed to remain balanced. Moments of stability occur during every step made by participant #3 (Fig 5a, upper graph), while the participant with a higher BBS score shows moments of stability only when turning and at the way back (Fig 5a, lower graph). Fig 5b shows the margin of stability in the medial-lateral direction. In the lower part of each sub-figure the maximum distance of the non-affected side to the *x*-axis of *ϕ*^*w*^ is presented and in the upper part of each sub-figure the maximum distance of the affected side to the same axis is presented. Margin of stability in the medial-lateral direction of participant #3 appeared to be more symmetrical while walking in a straight line compared to the turning phase (first two parts of the upper graph of Fig 5b). The margin of stability in the medial-lateral direction of participant #10 is asymmetrical for both phases. When walking in a straight line, this participant shows an asymmetry in the direction of the affected side and while turning the asymmetry is in the direction of the non-affected side (first two parts of the lower graph of Fig 5b).

### Metric evaluation

**Fig 6:**
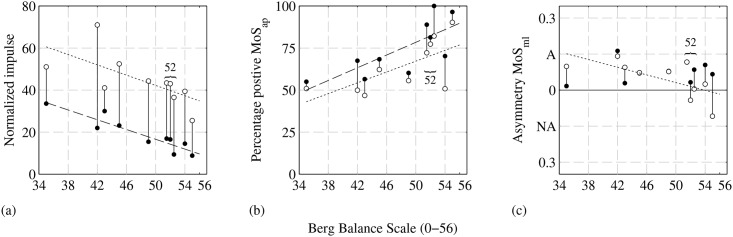
Dynamic gait metrics as a function of the individuals BBS score. Metric values are estimated for the walking and turning phase of the TUG test and per phase averaged over both trials of each participant. Filled bullets indicate the first straight line walking phases, open bullets indicate the turn phases. Filled and open bullets, corresponding to the same participant, are connected with a vertical line. Long and short dashed lines show the linear regression of metric values which are significantly correlated with individual BBS scores, respectively while walking in a straight line or when turning. (a) Normalized impulse as metric of walking efficiency. (b) Percentage of the phase the MoS_*ap*_ was positive, i.e., XCoM outside the (v)BoS. (c) Asymmetry lateral margin of stability, A = affected side, NA = non-affected side, additional data (i.e., range and standard deviation per participant) are available in S1 and S2 Figs. Some participants show an almost similar asymmetry value while walking in a straight line or when turning, therefore some filled bullets are printed behind the open bullets.

The efficiency of walking per TUG test phase is presented for each participant in Fig 6a (mean values and standard deviations of this metrics and all other metrics are presented in Table 2). the estimated normalized impulse evaluates velocity direction (Fig 4a) and size (Fig 4b). Participants with lower BBS scores, show a higher normalized impulse when walking in a straight line (filled bullets, *r* = −0.92, *p* < 0.001) or when turning (open bullets, *r* = −0.67, *p* = 0.035), indicating a less efficient walking pattern as a result of frequent accelerations/decelerations and frequent change of the direction of CoM velocity.

**Table 2:** Overview of all metrics averaged over all participants, during the first straight line walking phases and the turn phases.

Metric	Units	Straight—Mean (±SD)	Turn—Mean (±SD)
Normalized impulse	a.u.^1^	19.0 (±8.2)	44.8 (±11.9)
Percentage positive MoS_*ap*_	%	74.5 (±16.3)	63.8 (±15.5)
Asymmetry MoS_*ml*_	a.u.^1^	0.08 (±0.04)	0.05 (±0.08)
MoS_*ap*_			
Affected side	m	0.07 (±0.06)	0.06 (±0.04)
Non-affected side	m	0.06 (±0.06)	0.01 (±0.04)
MoS_*ml*_			
Affected side	m	0.26 (±0.03)	0.25 (±0.04)
Non-affected side	m	0.24 (±0.02)	0.22 (±0.02)

^1^Arbitrary units

All participants show more instability in the anterior-posterior direction (a positive MoS_*ap*_, Fig 5a) when walking in a straight line compared to the turning phase (see Fig 6b). Participants with lower BBS scores, show less frequent instability when walking in a straight line (filled bullets, *r* = 0.75, *p* = 0.014) or when turning (open bullets, *r* = 0.67, *p* = 0.034).

The Asymmetry MoS_*ml*_ of all participants when walking in a straight line (filled bullets) is to their affected side (see Fig 6c). In these cases, participants’ lateral margin of stability (as in Fig 5b) is on average larger at their affected side, than on their non-affected side, i.e., the CoM’ is closer to their non-affected side. No significant correlation was found between participants’ Asymmetry MoS_*ml*_ when walking in a straight line (filled bullets, *r* = 0.24, *p* = 0.50) and their BBS scores. When participants are turning, those with lower BBS scores show a comparable or increase of their Asymmetry MoS_*ml*_ to their affected side, while participants with higher BBS scores were able to decrease their Asymmetry MoS_*ml*_ value or even obtained an Asymmetry MoS_*ml*_ to their non-affected side (open bullets, *r* = −0.66, *p* = 0.037). Hereby it should be noted that all participants, although not specifically instructed, made a turn in a counterclockwise direction while performing the TUG test. Therefore in all cases, participants’ inner leg was their affected side. Less affected participants were able to increase their lateral margin of stability at their non-affected side, their CoM is moving more towards their affected leg, so they lean in while making a turn.

**Fig 7:**
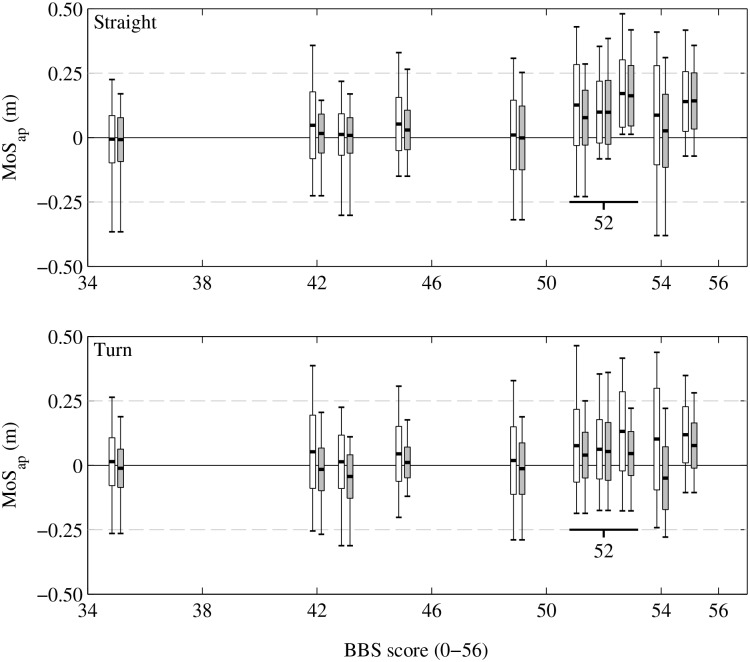
Anterior-posterior margin of stability MoS_*ap*_. MoS_*ap*_ distribution for all participants while standing on their affected side and non-affected side (grey), during the whole periods of straight line walking (upper graph) or the whole periods of turning (lower graph) of both TUG tests. Black vertical lines indicate MoS_*ap*_ ranges (minimum MoS_*ap*_ to maximum MoS_*ap*_, over multiple steps). Thick black markers indicates Mean MoS_*ap*_ values during the stance phases of the affected and non-affected side of multiple steps. Green and red areas indicate Mean ± SD MoS_*ap*_ values.

Fig 7 shows more detailed information for all participants on the distribution of MoS_*ap*_, while standing on their affected or non-affected side. When walking in a straight line (upper graph), participants show similar average MoS_*ap*_ values for their affected and non-affected side. Larger differences between the maximum MoS_*ap*_ values of both legs can be found. Participants’ maximum MoS_*ap*_ value on their affected side is larger than the maximum MoS_*ap*_ value on their non-affect side, except for participant #7 (BBS score = 52). When turning (lower graph), all participants show a large average MoS_*ap*_ at their affected side. When turning counterclockwise the traveled distance of the non-affected leg is larger and therefore the velocity of CoM is on average higher when standing on their affected side compared to their non-affected side. When turning, patterns seems to be similar, however participants #1, #2, #3, #5 and #9 (respectively, BBS scores are 35, 42, 43, 49 and 54) show on average a negative MoS_*ap*_ value when standing on their non-affected side. This means, these participants were stable when turning (on average), when standing on their non-affected side.

## Discussion

Presented system and analysis methods allow the assessment of walking balance and efficiency of walking during a structured daily life task. Previous assessment methods of balance during walking are mostly lab based and used for the evaluation of walking in a straight line [7, 11, 15, 20, 22, 23]. Although tested in a structured environment, measurements with the presented combination of instrumented shoes and data interpretation methods are no longer restricted to a lab. Where others developed instrumented TUG tests to demonstrate mobility characteristics that could not be quantified by simple visual analysis [29, 30], we additionally included and integrated kinematic parameters besides temporal and spatial parameters. Now, a first step is made that allows evaluation of walking balance for an unlimited number of steps during variable walking and other challenging walking conditions such as a TUG test. Data of straight line walking as well as turning can be assessed and result in objective and patient specific metrics. These metrics describe participants balance function on an activity level as well as on a level of body function, as these are described in the ICF. For example: walking velocity, describes balance function on an activity level, and the margin of stability, describes the ability to control the XCoM’ position relative to the edge of the BoS on a body function level. The metrics which describe walking balance on the level of body function may provide additional insight in the underlying stabilizing mechanisms (on the level of body function) of walking and potentially discriminate between changes in functional performance caused by recovery of control in the affected leg (restitution) or compensation in the non-affected leg (substitution / compensation).

In this study, ten stroke survivors with different BBS scores, completed a TUG test twice. Results differ for the walking and turning phases of the TUG test. Less affected participants show a more efficient walking pattern, i.e. smaller average normalized impulse values, when walking in a straight line or when turning. Furthermore, the XCoM of less affected participants is more frequently outside their BoS and therefore these participants are more frequently unstable during walking, like in normal walking. Metrics relate to participants clinically assessed balance function, but large inter and intra-patient variations of proposed metrics are present which cannot be evaluated using for instance the BBS test. Thereby, objective information on walking balance during functional walking is relevant information besides clinically assessed balance function. It allows the individual assessment of underlying mechanisms of balance control in a daily life setting and might explain any clinically assessed functional recovery by restitution of body function or compensation for the lack of restoration of body function.

Changes in walking velocity and walking direction are related to a less efficient walking pattern [24, 34]. The normalized impulse describes these changes in walking velocity and direction. More affected participants show larger normalized impulse values when walking in a straight line and when turning (see Fig 6a). A higher normalized impulse is the result of larger changes in walking direction and higher variations in walking velocity. Although the normalized impulse is correlated with BBS scores, large variations are present which are likely to be caused by different strategies used to complete the task. For instance, participant #1 who has a BBS score of 35, shows a smaller increase in normalized impulse when turning relative to walking in a straight line, compared to other participants. We noticed that this participant increased its turning radius when making the turn. By increasing the turning radius, the length of the walking path increases, the number of steps needed to complete the task increases, but the walking direction is more gradually changing while turning. The participant was able to remain a more constant walking velocity during this gradual change of walking direction, so there was less acceleration and deceleration which resulted in a smaller increase of the normalized impulse for this specific participant when turning.

The Asymmetry MoS_*ml*_ value is different for participants walking in a straight line compared to turning. When walking in a straight line, participants shift weight between their left and right leg. Generally, more affected stroke survivors show an unequal weight bearing towards their unaffected side [1]. However, no significant correlation was found between the asymmetry in medial-lateral margin of stability and participants’ BBS score when walking in a straight line. This might be caused by different foot placement strategies. Foot orientation, i.e., pointing a foot less or more outwards, may influence the lateral margin of stability and thereby the asymmetry in lateral margin of stability. Further research should focus on foot placement strategies related to the lateral margin of stability. When turning, compared to straight line walking, more affected participants show an increase of their Asymmetry MoS_*ml*_ towards their affected side. This is caused by a decrease of their lateral margin of stability at their non-affected side, so CoM’ is relatively moving towards their non-affected side. Less affected participants are able to shift their lateral margin of stability to or towards their non-affected side, so CoM’ is moving towards their affected side. These less affected participants appear to have more control and are more confident to move their CoM almost above their affected side when turning.

When turning, some participants reduce their average MoS_*ap*_ when standing on their non-affected side, such that their XCoM’ is mainly inside their (v)BoS. On average, these participants are dynamically stable when turning, so they can stop forward progression when turning. This will probably give these participants a higher level of confidence, knowing they can stop walking during a more complex task. During straight line walking MoS_*ap*_ is positive more frequent (see Fig 6b) and the average MoS_*ap*_ of almost all participants is positive for both sides (see Fig 7). In addition, the maximum MoS_*ap*_ of participants’ affected side is larger for almost all participants (see Fig 7). The MoS_*ap*_ value, that is the distance of XCoM’ to (v)BoS, is directly related to walking velocity as XCoM position depends on the velocity and position of CoM [21]. Larger maximum MoS_*ap*_ distances at participants’ affected side are related to higher CoM velocities when standing on the affected side. These higher velocities of CoM are generated by stronger push-offs of participant’s non-affected side, which reduces the stance times at the affected side [15, 24, 35]. An example of this varying velocity can be seen in participant #3 (upper graph of Fig 4b), the maximum magnitude of the walking velocity varies. Those participants who are confident enough and have good control on their healthy side may use this variation to reduce their TUG time by compensation. They compensate their lacking push-offs at their affected side by using their ability to accelerate and decelerate mainly by their non-affected side.

A few limitations of the presented work should be acknowledged. First, participants’ history of falls is unknown. A history of falls might explain some variation in metric values. Those with a history of falling may walk more carefully by decreasing their walking speed, especially if they are performing more complex tasks [13, 23]. Second, while completing the TUG test, all participants were turning counterclockwise around the turn marker towards their affected side. The direction of the turn was a choice of the participant, no instructions on turning during the TUG test were given. According to Faria and colleagues, there is no relation between the hemiparesis of stroke survivors and results of the TUG test while turning towards their affected or the non-affected side [13]. However, it has been reported that the occurrence of falls in stroke survivors is more frequent toward their affected side [5]. Therefore, in future experiments, it can be of interest to evaluate differences in turning performance and strategies of turns towards stroke survivors’ affected as well as non-affected side. Third, included participants vary in gender, age (range 47 to 75 years old) and time post stroke (range 0.7 to 7.4 years), however all included participants have a left affected side and almost all have a right dominant side. A more heterogeneity in affected and dominant sides, together with the evaluation of clockwise and counterclockwise turns, may reveal patient specific differences in turning strategies and may result in specific guidance of rehabilitation practice for left or right side affected stroke survivors. Fourth, any ground contact other than the shoes (e.g., using a walking aid, sitting in a chair, leaning on something) results in an incomplete/incorrect measurement of forces and so influences the kinetic reconstruction and all other variables. Presented methods can only be used in those who are able to walk without walking assistance. Any kinetic reconstructions of transitions or walking using walking aids can only be made when additional equipment is used. Finally, moments of turning were manually selected based on reviewing the data, which may introduce small errors while comparing both walking phases. An automated turning detection algorithm could be more consistent and more accurate [29].

In addition to presented work, future research should evaluate clinical relevance of presented metrics for the monitoring, training and coaching of the individual patient during rehabilitation in a daily life setting. A longitudional designed study may demonstrate participant specific changes of metrics over time. Results may be used to objectively explain any changes in clinically assessed balance function of the individual stroke survivor, whether it is restoration of body function or learned compensatory strategies to overcome lack of restoration of body function. For example, if a decrease in time to complete a TUG test is related to respectively an increase in MoS_*ml*_ of both sides or only a increase in MoS_*ml*_ at the affected side. Furthermore, there should be a focus on the development of a small and well integrated sensing system [18]. Current sensing structure is limited to the shoes, which is a good basis for developing an unobtrusive sensing system in user’s own shoes. By further integration of the instrumented shoes and the additional ultrasound system, synchronization problems can be solved. Besides these design suggestions, algorithms should also be improved to make the system even more applicable for evaluation of walking in daily life. A rough activity classifier allows automated analysis of different walking patterns such as continuous and variable walking [16], but could also prevent analysis of incomplete kinetic reconstructions when a participant is leaning towards something or is seated.

## Conclusions

We demonstrated a method to assess walking balance of stroke survivors, during challenging walking conditions such as turning. Although tested in a structured environment, the system design and evaluation methods are not restricted to lab based measurements and in principle allow the evaluation of walking balance in a daily life setting. This objective evaluation of walking balance and efficiency may in future allow the clinician to distinguish between improvement by restoration of body function or recovery by learned compensation strategies in individual patients.

## Supporting Information

S1 File Processed data files. A compressed folder containing three files. First file is a text file containing additional information on opening the data file. Second file is a MATLAB^®^ data file which contains all study data: kinematic and kinetic reconstructions, and participant information. All data is within one structure, including data for all participants performing two times a TUG test and overall participant characteristics as in Table 1. Data are structured per patient and per TUG test and includes: fused inertial and ultrasound sensor data into shoe positions, forces measured per force sensor, processed kinematic and kinetic data: CoM and XCoM positions and variables as plotted in Figs 4 and 5. Third file is a PDF which explains the data structure of the data file.
(ZIP)

S1 Fig Distribution of asymmetry in lateral margin of stability. Distribution of asymmetry in lateral margin of stability of all participants during periods of straight line walking (filled bullets) and during periods of turning (open bullets) of both TUG tests. A = affected side, NA = non-affected side. Black vertical lines indicate Asymmetry MoS_*ml*_ ranges (minimum Asymmetry MoS_*ml*_ to maximum Asymmetry MoS_*ml*_ over multiple steps). Thick black markers indicates Mean Asymmetry MoS_*ml*_, values over multiple steps, as in Fig 6c. Areas indicate Mean ± SD Asymmetry MoS_*ap*_ values.
(TIF)

S2 Fig Medial-lateral margin of stability MoS_*ml*_. Medial-lateral margin of stability MoS_*ml*_. MoS_*ml*_ distribution for all participants while standing on their affected side (green) and non-affected side (red) leg, during the whole periods of straight line walking (upper graph) or the whole periods of turning (lower graph) of both TUG tests. Black vertical lines indicate MoS_*ml*_ ranges (minimum MoS_*ml*_ to maximum MoS_*ml*_, over multiple steps). Thick black markers indicates Mean MoS_*ml*_ values during the stance phases of the affected and non-affected side of multiple steps. Green and red areas indicate Mean ± SD MoS_*ml*_ values.
(TIF)
