# Supplementary material for: Analysis of Balance during Functional Walking in Stroke Survivors
Source: PLoS One. 2016 Nov 17;11(11):e0166789. doi: 10.1371/journal.pone.0166789 (PMC5113974; doi:10.1371/journal.pone.0166789)
Supplement: S1 File — A compressed folder containing three files. First file is a text file containing additional information on opening the data file. Second file is a MATLAB® data file which contains all study data: kinematic and kinetic reconstructions, and participant information. All data is within one structure, including data for all participants performing two times a TUG test and overall participant characteristics as in Table 1. Data are structured per patient and per TUG test and includes: fused inertial and ultrasound sensor data into shoe positions, forces measured per force sensor, processed kinematic and kinetic data: CoM and XCoM positions and variables as plotted in Figs 4 and 5. Third file is a PDF which explains the data structure of the data file. (ZIP) [file pone.0166789.s001.zip › DATAreference.pdf]

	Type	Size
**DATA.**		
→ version	string	
→ comment	string	
↘ **information.**		
→ articleName	string	
→ publisher	string	
→ authors	string	
→ DOI	string	
↘ **Participant(i).**		
→ sampleRate	double	(1 x 1)
↘ **info.**		
→ Participant_ID	double	(1 x 1)
→ Gender	string	
→ DominantSide	string	
→ AffectedSide	string	
↘ **Age.**		
→ unit	string	
→ value	double	(1 x 1)
↘ **PostStroke.**		
→ unit	string	
→ value	double	(1 x 1)
↘ **Weight.**		
→ unit	string	
→ value	double	(1 x 1)
→ MeasuredWithShoe_unit	string	
→ MeasuredWithShoe_value	double	(1 x 1)
↘ **Height.**		
→ unit	string	
→ value	double	(1 x 1)
↘ **BBS.**		
→ name	string	
→ unit	string	
→ value	double	(1 x 1)
↘ **TUG.**		
→ name	string	
→ unit	string	
→ value	double	(1 x 2)
↘ **TMW.**		
→ name	string	
→ unit	string	
→ value	double	(1 x 1)

i = participant ID (1 to 10)

		Type	Size
**DATA.**			
↘ **Participant(i).**			
↘ **TUG_DATA(t).**			
→	TUG_ID	double	(1 x 1)
↘ **Straight. or Turn.**			
↘ **PAR.**			
→	TurnAngle	double	(n x 1)
→	vCoM	double	(n x 3)
→	aCoM	double	(n x 3)
→	GroundContactLeft	double	(n x 1)
→	GroundContactRight	double	(n x 1)
→	MoS_AP	double	(n x 1)
↘ **MoS_ML.**			
→	Right	double	(n x 1)
→	Left	double	(n x 1)
↘ **POS.**			
→	CoM	double	(n x 3)
→	XCoM	double	(n x 3)
↘ **Left. or Right.**			
→	Heel	double	(n x 3)
→	Toe	double	(n x 3)
→	ForceSensorHeel	double	(n x 3)
→	ForceSensorForefoot	double	(n x 3)
→	CoP	double	(n x 3)
↘ **F.**			
↘ **Left. or Right.**			
→	Heel	double	(n x 3)
→	Forefoot	double	(n x 3)
→	GRF	double	(n x 3)

i = participant ID (1 to 10)
t = trial number (1 or 2)

n = number of samples
