# Supplementary figures and images for: Analysis of Balance during Functional Walking in Stroke Survivors
Source: PLoS One. 2016 Nov 17;11(11):e0166789. doi: 10.1371/journal.pone.0166789 (PMC5113974; doi:10.1371/journal.pone.0166789)

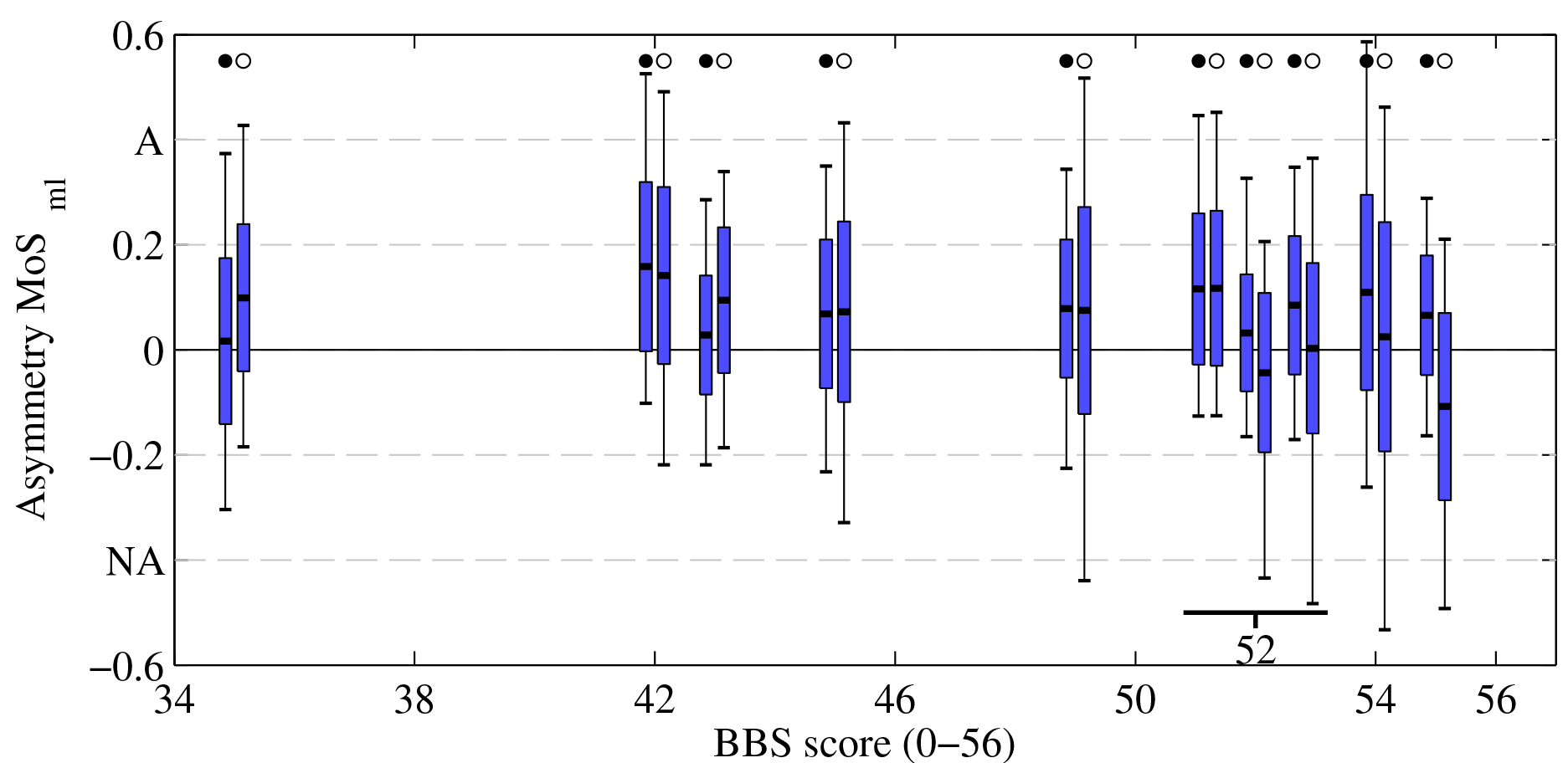

Supplement: S1 Fig — Distribution of asymmetry in lateral margin of stability of all participants during periods of straight line walking (filled bullets) and during periods of turning (open bullets) of both TUG tests. A = affected side, NA = non-affected side. Black vertical lines indicate Asymmetry MoSml ranges (minimum Asymmetry MoSml to maximum Asymmetry MoSml over multiple steps). Thick black markers indicates Mean Asymmetry MoSml, values over multiple steps, as in Fig 6c. Areas indicate Mean ± SD Asymmetry MoSap values. (TIF) [file pone.0166789.s002.tif]

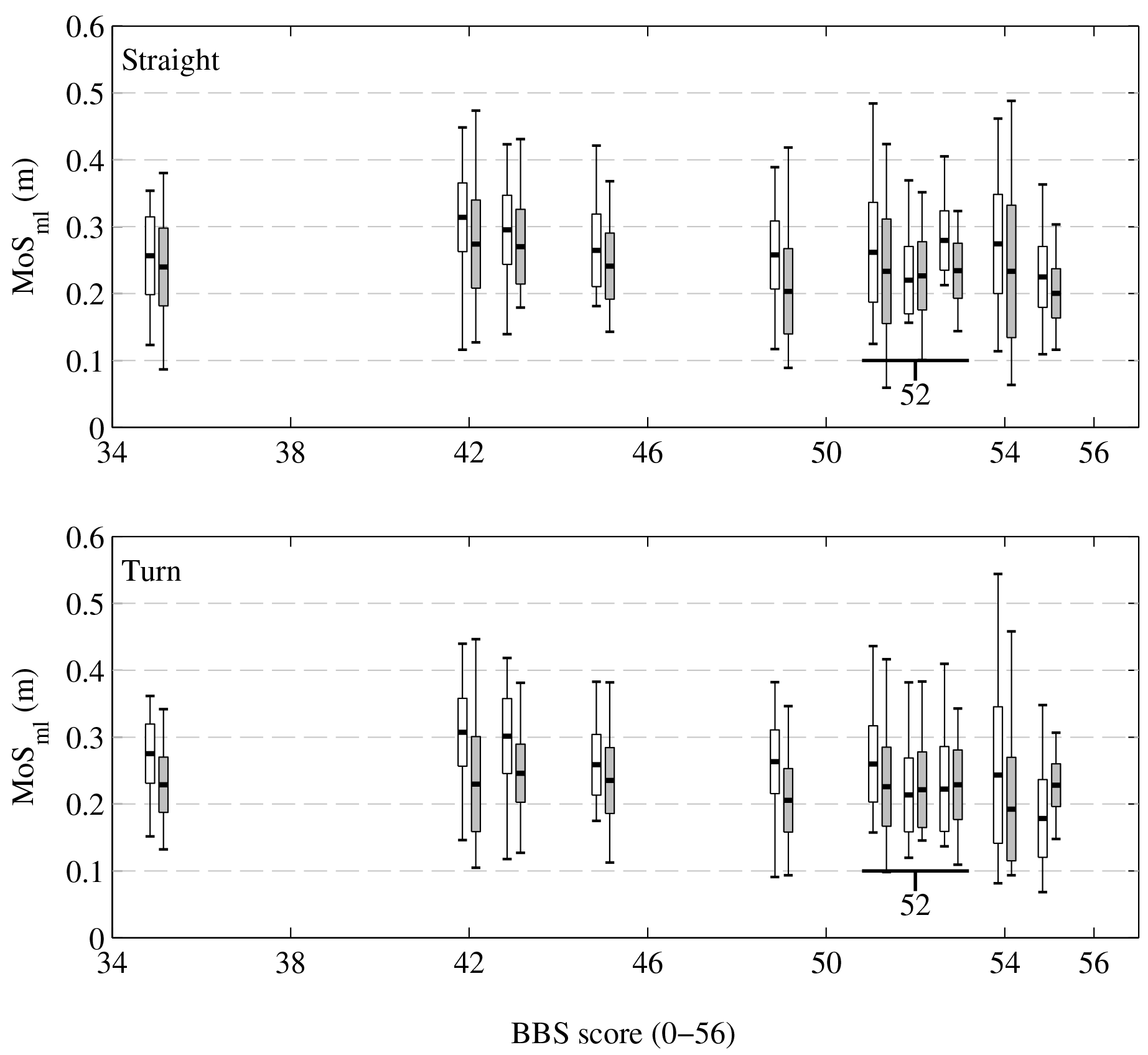

Supplement: S2 Fig — Medial-lateral margin of stability MoSml. MoSml distribution for all participants while standing on their affected side (green) and non-affected side (red) leg, during the whole periods of straight line walking (upper graph) or the whole periods of turning (lower graph) of both TUG tests. Black vertical lines indicate MoSml ranges (minimum MoSml to maximum MoSml, over multiple steps). Thick black markers indicates Mean MoSml values during the stance phases of the affected and non-affected side of multiple steps. Green and red areas indicate Mean ± SD MoSml values. (TIF) [file pone.0166789.s003.tif]
